# Novel biomarkers for early prediction of sepsis-induced disseminated intravascular coagulation in a mouse cecal ligation and puncture model

**DOI:** 10.1186/1476-9255-10-7

**Published:** 2013-03-05

**Authors:** Jingchun Song, Dunzhong Hu, Chao He, Tao Wang, Xuefeng Liu, Linhao Ma, Zhaofen Lin, Zili Chen

**Affiliations:** 1 Department of Emergency and Critical Care Medicine, Shanghai Changzheng Hospital, The Second Military Medical University, Shanghai 200003, China; 2 Department of Emergency and Critical Care Medicine, the 94th Hospital of PLA, Nanchang, Jiangxi Province 330002, China

**Keywords:** Protein microarray, Disseminated intravascular coagulation, Mouse, Cecal ligation and puncture, Platelet-derived factor

## Abstract

**Introduction:**

The objective of this study was to identify biomarkers of sepsis-induced disseminated intravascular coagulation (DIC) among platelet-derived factors using biotin label-based custom protein microarray technology in a mouse cecal ligation and puncture (CLP) model.

**Methods:**

KM mice were randomized into sham-operated and CLP groups. Blood samples were obtained immediately and at 1 h, 2 h, 6 h, 12 h, 24 h, 48 h and 72 h after establishment of the CLP for platelet count, coagulation assay and blood chemistry. Lung and mesentery tissues were examined histologically at all corresponding time points, looking for microthrombus formation. Serial protein microarray analysis was performed to detect platelet-derived factors.

**Results:**

The survival rate 72 h post-CLP was 15%, but there was no mortality among the sham-operated mice. Compared with the sham group, the platelet count (*n* = 5, *p* < 0.05), fibrinogen concentration (*n* = 5, *p* < 0.05) and alanine aminotransferase level of the CLP group began to decrease significantly at 6 h post-CLP. Significant prolongation of prothrombin time (*n* = 5, *p* < 0.05) and activated partial thromboplastin time (*n* = 5, *p* < 0.05) and elevation of D-dimer (*n* = 5, *p* < 0.05) occurred after 6 h post-CLP. On histology, microthrombus formation in lung and mesentery tissue was observed in the CLP groups 6 h post-CLP and had become significant and extensive 12 h post-CLP (*n* = 5, *p* < 0.05). On protein microarray analysis and ELISA, thrombospondin (TSP), tissue inhibitor of metalloproteinase 1 (TIMP-1) and thymus chemokine-1 (TCK-1) all increased during the first 2 h post-CLP, then remained at a higher level than in the sham group for 72 h post-CLP (*n* = 5, *p* < 0.05).

**Conclusions:**

TSP, TIMP-1 and TCK-1 are elevated in the early stage of sepsis-induced DIC in a mouse CLP model and may be considered early markers for sepsis-induced DIC.

## Introduction

Severe sepsis remains a serious problem worldwide, with intensive care unit death rates ranging between 30% and 70% even under the best of care [1-4]. Sepsis generally results from the release of cytokines and the activation of plasma protein cascades such as the coagulation and fibrinolytic systems [5-7]. Disseminated intravascular coagulation (DIC) is a complex syndrome characterized by activation of the haemostatic and fibrinolytic systems with increasing loss of localization and compensated control [8]. DIC is a common complication of sepsis and is associated with a poor outcome [9-11]. Within this process, activation of coagulation, inhibition of fibrinolysis and consumption of coagulation inhibitors lead to a procoagulant state resulting in inadequate fibrin removal and fibrin deposition in the microvasculature [12]. Eventually, diffuse obstruction of the microvascular bed gives rise to progressive organ dysfunction, such as renal insufficiency, acute respiratory distress syndrome, hypotension and circulatory failure. Because of consumption of coagulation factors and the interference of fibrin degradation products, diffuse bleeding may occur [13].

Early warning signs of DIC are often nonspecific and subtle, but the clinical course may be alarmingly fulminant, leading to death within days of onset. Thus, early identification of sepsis-induced DIC is a major diagnostic problem. In 2001, an International Society of Thrombosis and Haemostasis (ISTH) subcommittee divided DIC into two stages: non-overt DIC with a stressed but compensated haemostatic system; and overt DIC with a stressed and uncompensated haemostatic system [14]. On this basis, a scoring system for overt DIC was proposed by the ISTH, using which overt DIC can be diagnosed in 25% in patients with sepsis [15,16]. However, these diagnostic algorithms remain far from gold standard [17,18].

Much basic and clinical research has been focused on the crossroads of coagulation and inflammatory pathways that is important in the pathogenesis of sepsis and DIC [19,20]. As key factors in coagulation and immune mediators, platelets not only “plug the leak” in a damaged vessel, but also secrete chemokines and proinflammatory cytokines essential for host defense against microbial infection [21-23]. Regarding the pathogenesis of sepsis-induced DIC, these platelet-derived factors warrant research into their use for early diagnosis and prediction of outcome [24,25]. To identify valuable diagnostic biomarkers of sepsis-induced DIC among various platelet-derived factors, a protein microarray kit was customized for use with a mouse cecal ligation and puncture (CLP) model. Platelet-derived factors were detected by protein microarray analysis at different times up to 72 h after establishment of the CLP model. We report here the development of DIC in the experimental mice and the identification biomarkers predictive of the early stage of sepsis-induced DIC.

## Materials and methods

### Animals

Male Kunming mice (3–4 weeks of age, 19–21 g/body weight) were obtained from the Experimental Animals Center of the Second Military Medical University (Shanghai, China) and maintained under pathogen-free conditions at a room temperature of 23±3°C and air humidity of 55±15% in a 12 h light/12 h dark cycle. The experimental protocols were approved by the Committee of Animal Experimentation of the Second Military Medical University.

### Anesthesia and CLP Surgery

Mice were subjected to the CLP procedure [26]. They were not fasted the night before surgery. Briefly, the mice were anesthetized with 2% sodium phenobarbital (50 mg/kg i.p.) (Sigma, USA). Following a 1 cm midline abdominal incision, the cecum was exposed and ligated at approximately two-thirds of its length from the distal tip, to maintain intestinal continuity. The cecum was then punctured twice with a 22-gauge needle and a small amount of its contents was expressed through the punctures. A sham operation (laparotomy and exposure of the cecum without any further manipulation) was performed as a control. The incision was closed and 1 mL of normal saline was administered subcutaneously. After surgery, the animals had unrestricted access to food and water. For mice that died during the night, the time of death was recorded as the time at which they were observed in the early morning. Samples were taken from those that survived and the mice sacrificed at sampling time points.

### Time course after CLP challenge

Mice were divided into two groups and subjected to either CLP or a sham operation (*n* = 5). Blood was obtained from the inferior vena cava for platelet count, coagulation assay and biomarker analysis at 0, 1, 2, 6, 12, 24, 48 or 72 h after the treatment. They were then sacrificed and autopsied for gross and microscopic evidence of DIC.

### Blood chemistry

Blood samples were centrifuged at 3000 *g* for 10 min to obtain serum. Alanine aminotransferase (ALT) and creatinine were measured using an autoanalyzer (Sysmex DRI-CHEM 3500; Fuji, Japan).

### Histology of lung and mesentery tissue

Lung and mesentery specimens were fixed in 10% buffered formalin, processed by standard techniques and embedded in paraffin. Cross-sectional cuts 3 μm thick were taken from the middle zones of the lungs and mesenteries [27]. The sections were stained with hematoxylin and eosin for histopathology and analysis of fibrin deposition, and examined with a light microscope (Zeiss Axioscop 40; Zeiss) by a pathologist who was blinded to the experimental groups. Ten high power fields were observed (×400), and digital images were obtained with a digital camera (Nikon 4500; Nikon Tokyo, Japan) and archived.

### Coagulation assay

Prothrombin time (PT), activated partial thromboplastin time (aPTT) and plasma fibrinogen were measured in an automated coagulometer (Sysmex CA-7000; Fuji, Japan). Platelet count was measured using an automatic blood cell counter (Advia 2120; Siemens, Germany). D-dimer was measured by enzyme-linked immunosorbent assay (R&D Systems, USA).

Laboratory diagnosis of DIC in the mice [28] required the presence of the following abnormalities: (1) PT ≥3 s more than that of the controls and/or aPTT ≥5 s above the upper limit of normal; (2) an absolute decrease in plasma fibrinogen concentrations >25%; (3) an absolute decrease in platelet count; and (4) positive hematoxylin and eosin staining for intravascular fibrin formation on post mortem tissue sections. Clinically, DIC in the mice was recognized by spontaneous epistaxis and bleeding at multiple sites and by evidence of gross internal hemorrhage at autopsy.

### Flow cytometry

Platelet activity was assessed using platelet activation markers (CD62P and CD63). Briefly, blood sample containing sodium citrate was centrifuged for 15 minutes at 1,500 rpm at room temperature. The samples were incubated with saturating concentrations of phycoerythrin (PE)-labeled antibodies (eBioscience, San Diego, CA, USA) against CD62P and CD63 with fluorescein isothiocyanate-labeled antibodies against CD61 for 30 minutes at room temperature in the dark. For control experiments, platelets were incubated with PE-coupled unspecific mouse IgG1 (eBioscience, San Diego, CA, USA) with the same ratio and concentration of fluorochrome-to-protein as specific IgG.

After immuno-labeling, the samples were analyzed by flow cytometry (BD FACSAria™, USA). Forward light scatter and CD61 expression were used for platelet identification. Platelet-bound anti-CD62P and anti-CD63 antibodies were determined by analyzing 10,000 platelets for PE-positive fluorescence.

### Biotin label-based cytokine and chemokine microarray assay

A custom mouse cytokine array kit (RayBiotech, USA) was purchased. The array spotted the membrane with 50 specific antibodies against cytokines and chemokines per our request. For the assay, we followed the instructions precisely as stated by the manufacturer. Briefly, membranes were placed in an eight-well tissue culture tray and incubated with 2 mL of blocking buffer at room temperature for 30 min. After decanting the blocking buffer from each container, 1 mL of sputum supernatant from the sample was added and incubated overnight at 4°C. After decanting the samples, all membranes were washed three times with 2 mL of wash buffer I at room temperature with shaking (5 min per wash), followed by two washes with wash buffer II at room temperature with shaking (5 min per wash). One milliliter of 1:250 diluted biotin-conjugated antibodies was prepared and incubated for 2 h at room temperature, and the washing steps were repeated as before. Two milliliters of 1:1000 diluted horse radish peroxidase-conjugated streptavidin was added, and the membranes were incubated for 2 h at room temperature, followed by additional washing. Spots were visualized using detection buffer C and D. Five hundred liters of a detection buffer C and 500 L of detection buffer D were mixed, loaded onto the membranes to cover the entire surface, and incubated for 5 min. The membranes were then covered in plastic wrap and exposed to radiographic film (Kodak X-Omat; Kodak; USA) for 20 min; the signal was detected using film developer. Each film was scanned (CanoScan, Japan) into an image-processing and analysis program (Image J, NIH Image for Windows, version 1.44), and spots were digitized into pixel densities. The densities were exported into spreadsheet software (Raybio Analysis Tool; Excel; Microsoft Corp) and the background intensity was deducted before analysis. The data were normalized to the values for positive controls provided by the manufacturer as 100%.

### ELISA

Blood samples were kept standing for 15 min until coagulation. After high-speed centrifugation (3000 rpm, 20 min), the supernatant was collected, aliquoted and stored at −70°C for analysis. The concentrations of thrombospondin (TSP), tissue inhibitor of metalloproteinase 1 (TIMP-1) and thymus chemokine-1 (TCK-1) were measured using specific ELISA kits (RayBiotech, USA) following the manufacturer’s protocols.Standard curves and regression equations were calculated based on measurements of standard samples, and the cytokine concentrations in the serum samples were calculated according to the regression equations.

### Statistical analysis

Results are presented as the mean ± standard error of the mean. When the data were normally distributed, Bartlett’s test was used to test variance equality. Survival curve data were analyzed using the Kaplan–Meier method and Wilcoxon’s test with SPSS 17.0 for Windows (SPSS Inc., IL, USA). Platelet count, blood chemistry findings, coagulation assay results, platelet activity, biomarker analysis and ELISA at various time points were compared between the CLP and sham groups by analysis of variance using a post hoc LSD method when the variances were equal. A one-way approximate *F* test was used for comparison when the variances were not equal. Lung and mesentery histology data were analyzed using Fisher’s test. *P*-values <0.05 were considered statistically significant.

## Results

### Mortality following CLP

**Figure 1:**
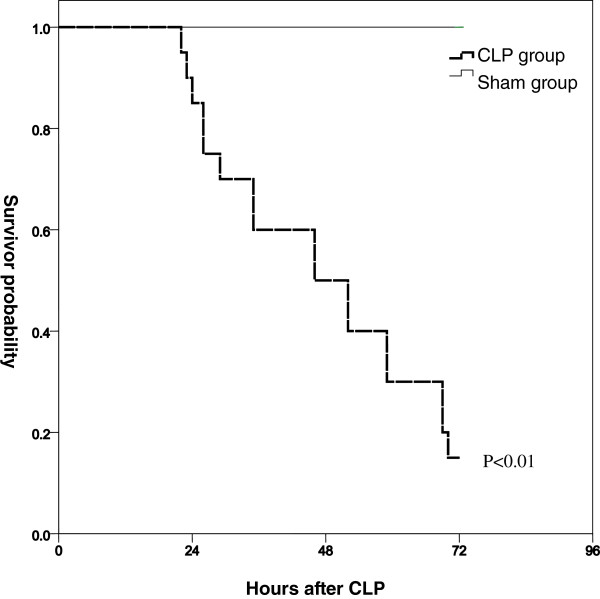
**Survival rate after cecal ligation and puncture (CLP).** The CLP procedure resulted in significantly greater mortality than a sham operation. CLP group, *n* = 20; sham group, *n* = 10. *p* < 0.01 vs. sham group (Wilcoxon’s test).

Twenty mice subjected to CLP and ten sham mice were observed for mortality. The survival rate at 72 h post-CLP was 15%, but there was no mortality among the sham mice (*p* < 0.01, Figure 1). This model was designed to achieve a high incidence of DIC; thus, this high mortality was expected.

### Development of DIC

**Figure 2:**
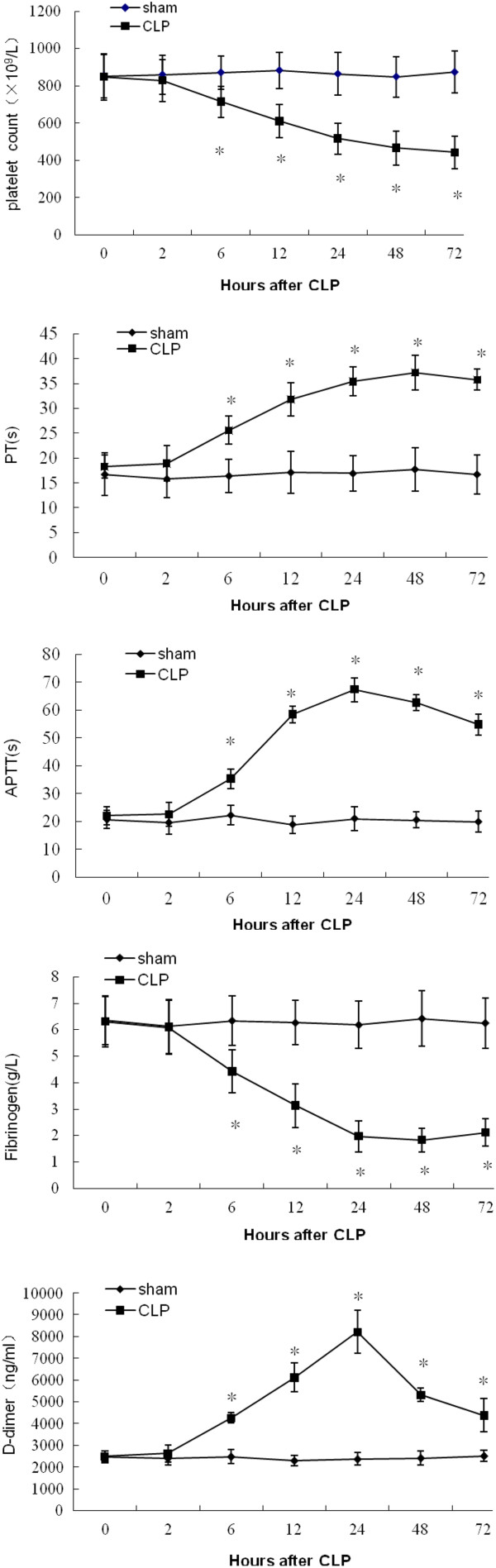
Blood markers of disseminated intravascular coagulation in cecal ligation and puncture (CLP) group and sham groups. ^*^Significant difference compared with sham group (*n* = 5, *p* < 0 .05).

The CLP model successfully induced DIC, as shown by changes in blood markers for DIC. Platelet count, PT, aPTT, fibrinogen and D-dimer results are shown in Figure 2. Compared with the sham group, the platelet count (*n* = 5, *p* < 0.05) and fibrinogen concentration (*n* = 5, *p* < 0.05) of the CLP group began to decrease significantly at 6 h post-CLP. Significant prolongation of PT (*n* = 5, *p* < 0.05) and aPTT (*n* = 5, *p* < 0.05) and elevation of D-dimer (*n* = 5, *p* < 0.05) also occurred from 6 h after CLP.

**Figure 3:**
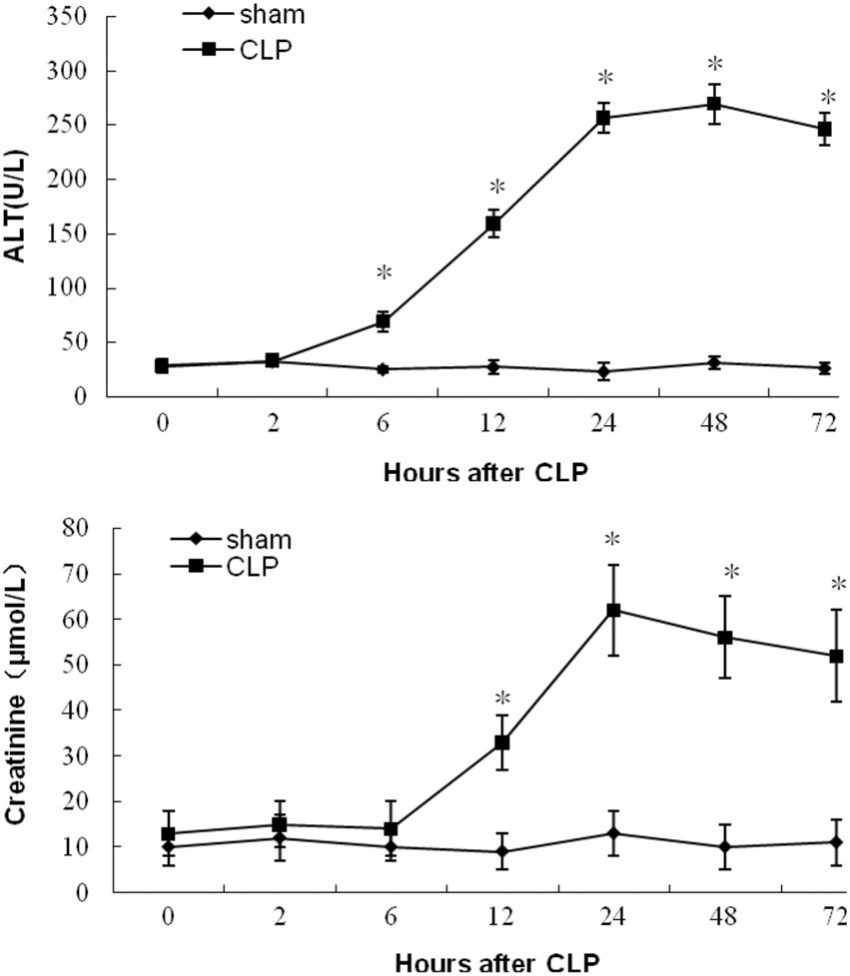
Blood chemistry in cecal ligation and puncture (CLP) group and sham group. ^*^Significant difference compared with sham group (*n* = 5, *p* < 0 .05).

Blood chemistry indicated that several organ damage markers were elevated above the sham values, including ALT and creatinine (Figure 3). The ALT of the CLP group was increased significantly 6 h post-CLP compared with the sham group (*n* = 5, *p* < 0.05). Significant elevation of creatinine was observed in the CLP group 12 h post-CLP (*n* = 5, *p* < 0.05). All of the above markers began to change significantly at 6 h post-CLP.

### Histology of lung and mesentery tissue

**Figure 4:**
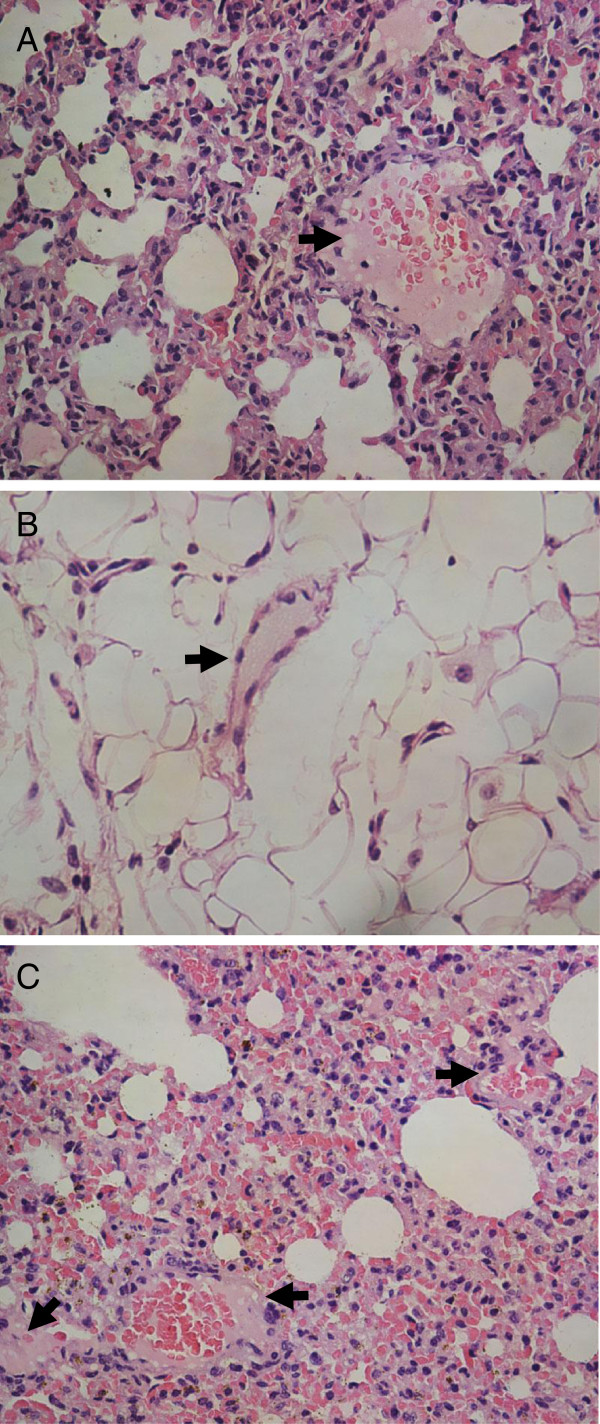
**Histological changes in mouse lung and mesentery tissue.** Samples were obtained 6 h post-cecal ligation and puncture (CLP) (hematoxylin and eosin; ×400). **A**: Mixed thrombus in lung tissue 6 h post-CLP. **B**: Fibrin thrombus in mesentery tissue 6 h post-CLP. **C**: Multiple thrombi in lung tissue 12 h post-CLP.

Histologic results for microthrombus formation in the CLP and sham groups are shown in Figure 4 and Table 1. In contrast to the sham group, neutrophil exudation and tissue swelling had occurred in lung and mesentery tissue 1 h post-CLP, but microthrombus formation was not detected (*n* = 5, *p* > 0.05). Signs of microthrombus had appeared 6 h post-CLP, and became obvious and extensive after 12 h (*n* = 5, *p* < 0.05). Simultaneously, alveolar consolidation, severe edema and tissue necrosis appeared. There were no pathological changes in the sham group.

**Table 1:** **Histology for microthrombus formation in lung and mesentery tissue from CLP and sham groups over time (*n *= 5)**

		**0 h**	**1 h**	**2 h**	**6 h**	**12 h**	**24 h**	**48 h**	**72 h**
Sham group	Lung	0	0	0	0	0	0	0	0
	Mesentery	0	0	0	0	0	0	0	0
CLP group	Lung	0	0	0	3^*^	5^*^	5^*^	5^*^	5^*^
	Mesentery	0	0	0	2^*^	4^*^	5^*^	5^*^	5^*^

^*^Significant difference compared with sham group (*n* = 5, *p* < 0 .05).

### Platelet activation status assessment by flow cytometry

**Figure 5:**
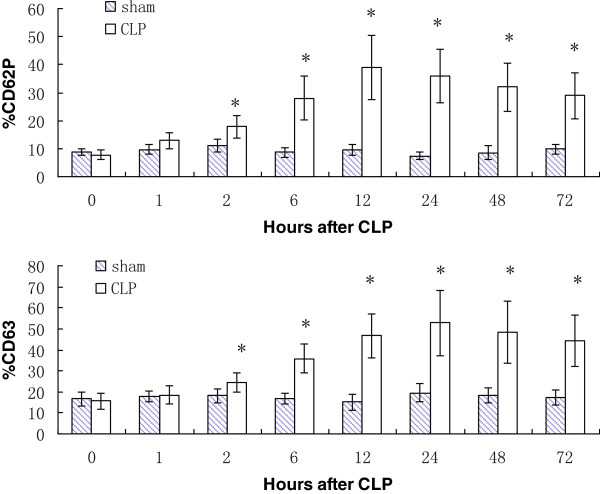
The percentage platelet surface expression of CD62P and CD63 by flow cytometry in CLP group and sham group. ^*^Significant difference compared with sham group (*n* = 5, *p* < 0 .05).

CD62P (P-selectin) is only expressed on the platelet surface after dense granule secretion, and CD63 is expressed on the platelet surface after the release of lysosomes or dense granules.So the percentage platelet surface expression of CD62P and CD63 are always quantified by flow cytometry to assess platelet activation status. In contrast to the sham group, the percentage platelet surface expression of CD62P and CD63 were significantly higher 2 h post-CLP (*n* = 5, *p* < 0.05, Figure 5).

### Protein microarray analysis

Given the importance of platelets in coagulation and as immune mediators, platelet-derived factors were chosen as biomarkers of the early stage of sepsis-induced DIC. To identify valuable diagnostic biomarkers, fifty platelet-secreted factors were selected for the custom biotin label-based mouse cytokine microarray assay kit. The name and location of each custom cytokine or chemokine spot are listed in Table 2. Based on the results of the coagulation assay, 6 h post-CLP was regarded as the early stage of sepsis-induced DIC.

**Table 2:** Biotin label-based custom protein microarray map

	**A**	**B**	**C**	**D**	**E**	**F**	**G**	**H**	**I**	**J**	**K**	**L**	**M**	**N**
1	POS1	POS2	POS3	Blank	neg	neg	Blank	bFGF	GM-CSF	MMP-9	Blank	IC-3	IC-2	IC-1
2	POS1	POS2	POS3	Blank	neg	neg	Blank	bFGF	GM-CSF	MMP-9	Blank	IC-3	IC-2	IC-1
3	Blank	Blank	Blank	TIMP-2	CCL-4	ICAM-2	Osteopo-rotegerin	TIMP-4	CCL-7	IFN-gamma	PDGF-C	TLR-1	CCR-3	IFN-gamma R1
4	Blank	Blank	Blank	TIMP-2	CCL-4	ICAM-2	Osteopo-rotegerin	TIMP-4	CCL-7	IFN-gamma	PDGF-C	TLR-1	CCR-3	IFN-gamma R1
5	CCR-4	IGFBP-3	PDGF-R beta	TLR-4	CD 11B	IL-1 alpha	PF-4	TPO	CD 40	IL-2	RAGE	TRAIL	CD 40L	IL-3
6	CCR-4	IGFBP-3	PDGF-R beta	TLR-4	CD 11B	IL-1 alpha	PF-4	TPO	CD 40	IL-2	RAGE	TRAIL	CD 40L	IL-3
7	PDGF-R alpha	TLR-2	RANTES	TREM-1	CXCR-4	IL-7	SCF	VEGF	DKK-1	CD 18	TFPI	Thymus Chmokine-1	Endostatin	Leptin
8	PDGF-R alpha	TLR-2	RANTES	TREM-1	CXCR-4	IL-7	SCF	VEGF	DKK-1	CD 18	TFPI	Thymus Chmokine-1	Endostatin	Leptin
9	TGF-beta 1	Tissue Factor	EPO	LIGHT	Thrombos-pondin	G-CSF	MMP-2	TIMP-1	Blank	Blank	Blank	POS 6	POS 5	POS 4
10	TGF-beta 1	Tissue Factor	EPO	LIGHT	Thrombos-pondin	G-CSF	MMP-2	TIMP-1	Blank	Blank	Blank	POS 6	POS 5	POS 4

A1, A2, B1, B2, C1, C2, L9, L10, M9, M10, N9 and N10 indicate positive controls. E1, E2, F1 and F2 indicate negative controls. L1, L2, M1, M2, N1, N2 indicate internal controls.

**Figure 6:**
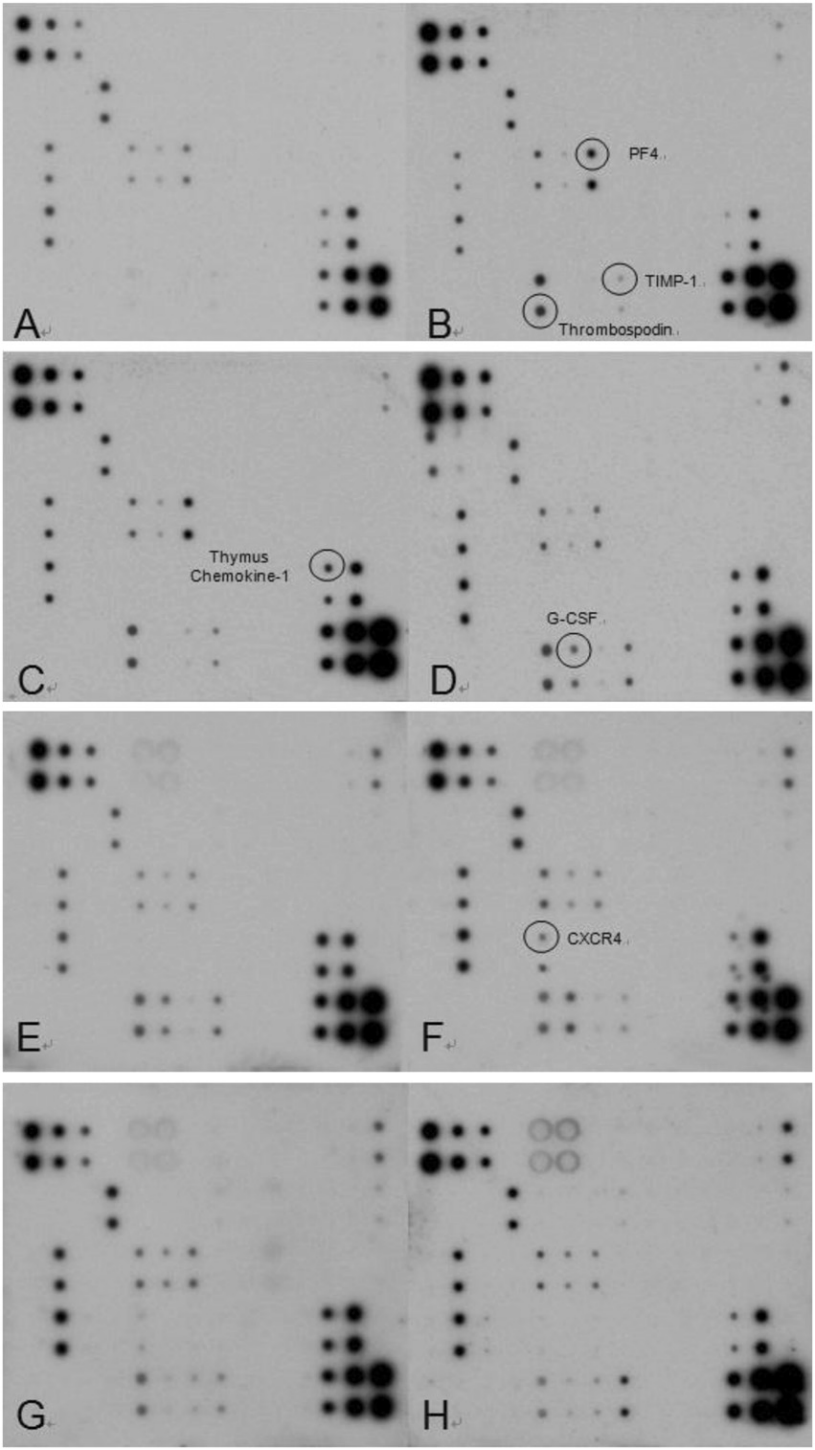
**Custom protein microarray analysis in cecal ligation and puncture (CLP) group and sham group. A**: Sham group. **B**: 1 h post-CLP group. **C**: 2 h post-CLP group. **D**: 6 h post-CLP group. **E**: 12 h post-CLP group. **F**: 24 h post-CLP group. **G**: 48 h post-CLP group. **H**: 72 h post-CLP group.

Compared with the sham group, thrombospondin (TSP) (E9, E10), platelet factor 4 (PF4) (G5, G6) and tissue inhibitor of metalloproteinase 1 (TIMP-1) (H9, H10) were obviously increased 1 h post-CLP (Figure 6A, Figure 6B). Thymus chemokine-1 (TCK-1) (L7, L8) was increased 2 h post-CLP (Figure 6C), granulocyte colony-stimulating factor (G-CSF) (F9, F10) had appeared 6 h post-CLP and C-X-C chemokine receptor type 4 (CXCR4) (E7, E8) had appeared 24 h post-CLP (Figure 6F). Therefore, TSP, PF4, TIMP-1and TCK-1 warrant evaluation as biomarkers of the early stage of sepsis-induced DIC.

**Figure 7:**
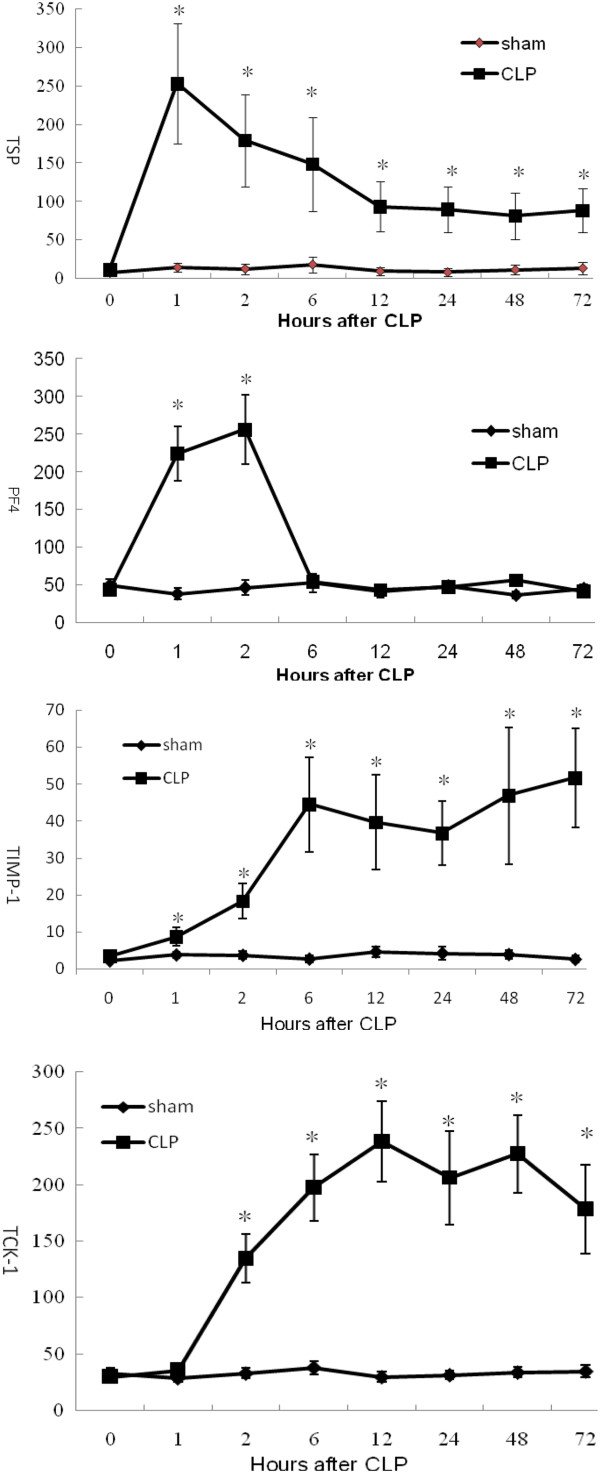
Protein microarray signal intensities of TSP, PF4, TIMP-1 and TCK-1 in cecal ligation and puncture (CLP) group and sham group. ^*^Significant difference compared with sham group (*n* = 5, *p* < 0.05).

**Figure 8:**
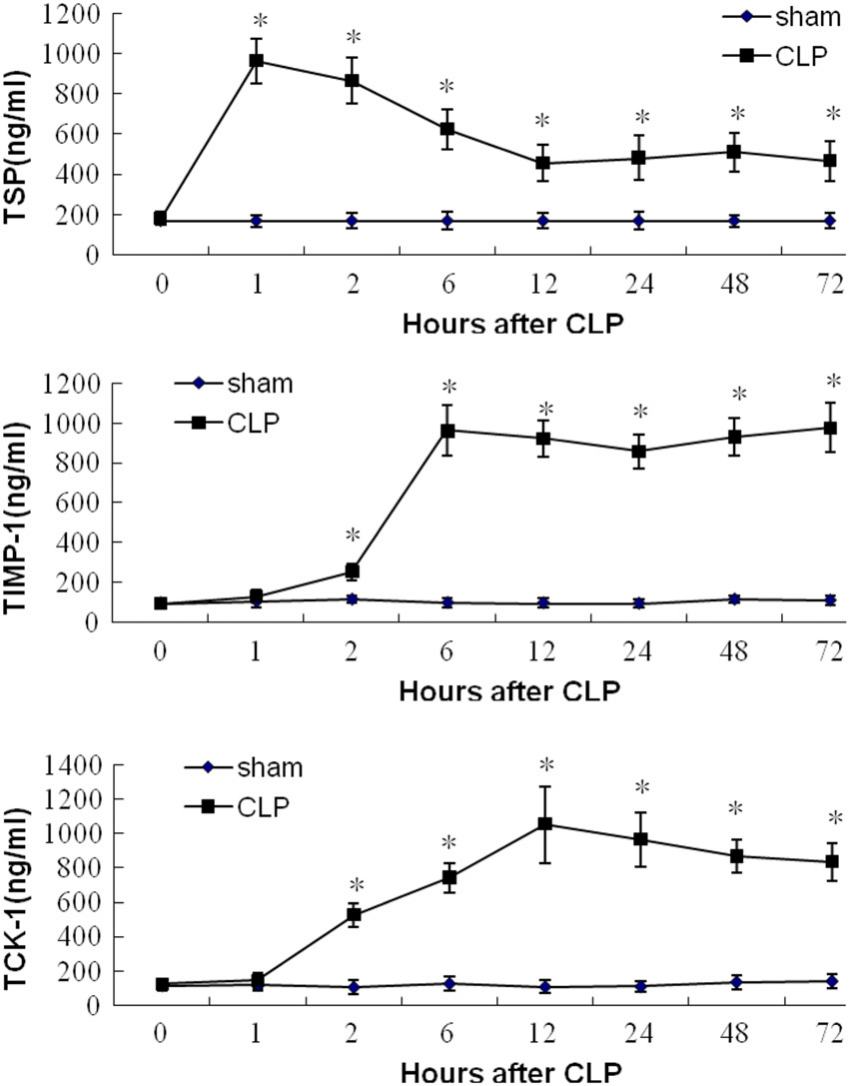
Serum concentrations of TSP, TIMP-1 and TCK-1 in cecal ligation and puncture (CLP) group and sham group by ELISA. ^*^Significant difference compared with sham group (*n* = 5, *p* < 0.05).

The protein microarray signal intensities of these cytokines and chemokines over time are summarized in Figure 7. TSP levels in the CLP group increased and peaked sharply at 1h post-CLP; they then decreased gradually until 72 h post-CLP, but remained significantly higher than in the sham group (*n* = 5, *p* < 0.05). PF4 levels in the CLP group were increased 1 h post-CLP, peaked at 2 h post-CLP (*n* = 5, *p* < 0.05), then decreased rapidly to the level of the sham group (*n* = 5, *p* > 0.05). TIMP-1 levels were also increased 1 h post-CLP, then continued to increase in the CLP group (*n* = 5, *p* < 0.05). TCK-1 levels in the CLP group were significantly increased 2 h post-CLP and remained higher than in the sham group until 72 h post-CLP (*n* = 5, *p* < 0.05). Somewhat differently, the serum levels of TIMP-1 measured by ELISA were also increased 2 h post-CLP significantly (*n* = 5, *p* < 0.05). Nevertheless, the serum levels of TSP, TIMP-1 and TCK-1 measured by ELISA have the similar change with the protein microarray signal intensities analysis (*n* = 5, *p* < 0.05, Figure 8).

## Discussion

As one of the most severe complications of sepsis, DIC is always associated with poor prognosis, multiply organ dysfunction and high mortality. Generally, clinical diagnosis of DIC relies on platelet count, coagulation assay, d-dimer and fibrinogen. However, when the results of such tests are positive, the best time for treatment had already passed. In much basic research, the difficulty of early prediction of sepsis-induced DIC is related to the large number of coagulation factors, chemokines and cytokines, and their complicated interactions. Platelet-derived factors are distinct from others because of their role at the crossroads of coagulation and inflammatory pathways such as the complement, contact phase, coagulation and fibrinolytic systems, host defense and antibacterial action [29,30]. Protein microarray technology allows us to study many molecules simultaneously and elucidate their interactions. A protein-detecting microarray comprises many different affinity reagents arrayed at high density. Each agent captures a target protein from a complex mixture, and the captured proteins are subsequently detected and quantified [31,32]. For the present study, a protein microarray kit was customized to serially detect fifty platelet-secreted factors in a mouse CLP model.

The CLP model imitates sepsis by creating a bowel perforation with leakage of fecal material into the peritoneal cavity. Since it was modified and popularized by Wichterman in 1980 [33], the CLP model has been considered the gold standard for sepsis research. Initially, CLP was not as widely used as lipopolysaccharide (LPS) as an inducer of DIC in animal models, probably because of its low reproducibility [34-36]. However, since its standardization by Rittirsch in 2009 [26], it has been possible to reproduce CLP exactly. Moreover, animals subjected to CLP simulate a true infectious septic state, and the decreased cardiovascular function and cardiac output induced by LPS are avoided [37]. In the CLP procedure used in the present study, the large ligation and needle size were necessary for high grade sepsis-induced DIC, and ensured 85% mortality at 72 h post-procedure. Development of DIC in the experimental mice was confirmed by the decreased platelet count and fibrinogen concentration, the prolongation of PT and aPTT, the elevation of D-dimer and the autopsy evidence of thrombosis in end-organs. The evolution of multiple organ dysfunction was corroborated by the increased ALT and creatinine. Six hours post-CLP was identified as the early stage of sepsis-induced DIC in the model, because all significant changes in clinical markers had occurred by this time. CD63 and CD62P (p-selectin) [38] are expressed on the platelet surface [39] and secreted into plasma [40] when platelets become activated. In resting platelets CD63 is stored in lysosomes and CD62P in a-granule. Therefore, the percentage platelet surface expression of CD62P and CD63 indicating platelet activation were significantly higher 2 h post-CLP. Simultaneously, the platelet-secreted factors TSP, PF4, TIMP-1 and TCK-1 changed significantly during the first 6 h post-CLP and were therefore regarded as meaningful biomarkers of the early stage of sepsis-induced DIC. G-CSF and CXCR4 were excluded because their levels in the experimental mice began to differ significantly after 6 h post-CLP.

TSP was first isolated from platelets that had been stimulated with thrombin, and was designated as a matricellular glycoprotein with multiple functions in many biological processes including angiogenesis, apoptosis and immune regulation [41,42]. PF4, also known as chemokine C-X-C motif ligand 4, is released from activated platelets during platelet aggregation and promotes blood coagulation [43]. TIMP-1, as natural inhibitor of matrix metalloproteinases has been shown to be involved in degradation of the extracellular matrix and to have an effect on the fibrinolytic system [44]. TCK-1 belongs to the family of CXC chemokines and has been shown to be a chemoattractant for pro-B cells engineered to express the CXCR2 receptor [45]. In the early stage of the mouse CLP model, TSP, PF4, TIMP-1and TCK-1 levels exhibited differing trends on protein microarray analysis. PF4 levels in the CLP group peaked 2 h post-CLP and decreased rapidly to the level of the sham group, which would made it difficult to capture abnormal PF4 levels in clinical practice. TSP levels in the CLP group increased and peaked sharply at 1 h post-CLP, then decreased but remained significantly higher than in the sham group. Both TIMP-1 and TCK-1 increased within 2 h post-CLP, then remained at higher levels than in the sham group until 72 h post-CLP. The long duration of the significant difference between experimental animals and controls indicates that TSP, TIMP-1and TCK-1 might be used to predict sepsis-induced DIC. The serum levels of TSP, TIMP-1 and TCK-1 measured by ELISA have proved that again.

Although elevations in cytokines/chemokines and their correlation with sepsis-induced DIC are meaningful observations, their relevance to the cause of DIC remains unclear. Further clinical trials will be required to validate the usefulness of TSP, TIMP-1 and TCK-1 in patients.

## Conclusions

We have demonstrated the development of DIC in experimental mice by means of decreased platelet count and fibrinogen concentration, prolongation of PT and aPTT, elevations of D-dimer and autopsy evidence of thrombosis in end-organs. By protein microarray analysis, we have identified TSP, TIMP-1and TCK-1 as biomarkers predictive of the early stage of sepsis-induced DIC.

## Abbreviations

ALT: Alanine aminotransferase; aPTT: activated partial thromboplastin time; CLP: Cecal ligation and puncture; CXCR4: C-X-C chemokine receptor type 4; DIC: Disseminated intravascular coagulation; G-CSF: Granulocyte colony-stimulating factor; ISTH: International Society of Thrombosis and Haemostasis; LPS: Lipopolysaccharide; PF4: Platelet factor 4; PT: Prothrombin time; TCK-1: Thymus chemokine-1; IMP-1: Tissue inhibitor of metalloproteinase 1; TSP: Thrombospondin.

## Competing interests

The authors declare that they have no competing interests.

## Authors’ contributions

All authors helped to draft the manuscript or critically revised it. All authors agree about the content of the paper and have read the manuscript and approved its submission to Critical Care. Furthermore, ZL and ZC participated in the design and coordination of the study. JS, DH and TW participated in data acquisition and finalized the manuscript. CH, XL and LM participated in statistical analysis.
